# Commercial glyphosate-based herbicides effects on springtails (Collembola) differ from those of their respective active ingredients and vary with soil organic matter content

**DOI:** 10.1007/s11356-020-08213-5

**Published:** 2020-03-09

**Authors:** Michael Maderthaner, Maureen Weber, Eszter Takács, Mária Mörtl, Friedrich Leisch, Jörg Römbke, Pascal Querner, Ronnie Walcher, Edith Gruber, András Székács, Johann G. Zaller

**Affiliations:** 1 Institute of Zoology, University of Natural Resources and Life Sciences Vienna (BOKU), Gregor Mendel Straße 33, A-1180 Vienna, Austria; 2 Agro-Environmental Research Institute, National Agricultural Research and Innovation Centre, Herman O. u. 15, Budapest, H-1022 Hungary; 3 Institute of Statistics, University of Natural Resources and Life Sciences Vienna (BOKU), Peter-Jordan-Straße 82, A-1190 Vienna, Austria; 4 ECT Oekotoxikologie GmbH, Böttgerstraße 2, 65439 Flörsheim, Germany

**Keywords:** Active ingredient, Adjuvants, Herbicides, Nontarget effects, Pesticides, Soil biota, Weed control

## Abstract

Glyphosate-based herbicides (GBH) are currently the most widely used agrochemicals for weed control. Environmental risk assessments (ERA) on nontarget organisms mostly consider the active ingredients (AIs) of these herbicides, while much less is known on effects of commercial GBH formulations that are actually applied in the field. Moreover, it is largely unknown to what extent different soil characteristics alter potential side effects of herbicides. We conducted a greenhouse experiment growing a model weed population of *Amaranthus retroflexus* in arable field soil with either 3.0 or 4.1% soil organic matter (SOM) content and treated these weeds either with GBHs (Roundup LB Plus, Touchdown Quattro, Roundup PowerFlex) or their respective AIs (isopropylammonium, diammonium or potassium salts of glyphosate) at recommended dosages. Control pots were mechanically weeded. Nontarget effects were assessed on the surface activity of the springtail species *Sminthurinus niger* (pitfall trapping) and litter decomposition in the soil (teabag approach). Both GBHs and AIs increased the surface activity of springtails compared to control pots; springtail activity was higher under GBHs than under corresponding AIs. Stimulation of springtail activity was much higher in soil with higher SOM content than with low SOM content (significant treatment x SOM interaction). Litter decomposition was unaffected by GBHs, AIs or SOM levels. We suggest that ERAs for pesticides should be performed with actually applied herbicides rather than only on AIs and should also consider influences of different soil properties.

## Introduction

Herbicides represent the globally most widely used class of pesticides in conventional agriculture; among them, glyphosate with 8.26 10^8^ kg year^−1^ is the most commonly used active ingredient (AI) (Benbrook 2016; Székács and Darvas 2012; Székács and Darvas 2018). Every pesticide consists of its AI (or a combination of several AIs) – here glyphosate – and a number of undisclosed adjuvants considered chemically inert and therefore biologically not relevant. Therefore, environmental risk assessments (ERAs) for pesticides mainly consider nontarget effects of AIs only. However, meanwhile, evidence is mounting for several glyphosate-based herbicides (GBH) that commercial formulations differ in their nontarget effects from AIs alone (Mesnage and Antoniou 2018; Mesnage et al. 2015; Mullin et al. 2016; Myers et al. 2016; Pereira et al. 2009; Simões et al. 2018; Székács and Darvas 2018). Several studies report adverse effects of either GBH or AIs on soil organisms and processes (Gill et al. 2018; Lins et al. 2007; Santadino et al. 2014; Zaller et al. 2018; Zaller et al. 2014). For Collembolans (*Folsomia candida*), it was shown that they avoided one GBH product even at the recommended dose but were unaffected by other GBH products (Niemeyer et al. 2018).

Each of the beforementioned studies has all been conducted under specific soil types and soil organic matter (SOM) levels. However, soil properties and Collembola activity (Potapov et al. 2017; Rendoš et al. 2016) and also absorption and half-life of pesticides are affected by SOM levels (Buch et al. 2016; Silva et al. 2019). To the best of our knowledge, no study experimentally tested interactive effects between GBH/AIs and SOM levels.

Springtails (Collembola) are important indicators of soil quality and sustainable land use and are among the most abundant soil arthropods with worldwide 6500 species reaching densities up to 100,000 individuals m^−2^ (Buchholz et al. 2017; Eisenhauer et al. 2011; Hopkin 1997). Springtails can be grouped into different life forms: Eu- and hemiedaphic species live mainly in the soils, while epedaphic species are active on the soil surface (Hopkin 1997). In soils, most Collembola feed on fungi or decaying plant material and can influence the growth of mycorrhizae and the development of fungal diseases (Filser et al. 2002; Hopkin 1997; Klironomos et al. 1992; Lartey et al. 1994). Further they act as prey for many arthropods such as spiders, mites and beetles (Frampton and van den Brink 2007; Pfingstmann et al. 2019). Collembola are also frequently used as test species in ERAs of pesticides (Bandow et al. 2014; Ockleford et al. 2017).

Studies investigating the effects of herbicides on Collembola showed no clear pattern, but rather a high variability of impacts. In a standard avoidance test, no significant effects of a GBH (product Montana, containing 30.8% AI) on the Collembola *F. candida* were found (Santos et al. 2012). Increased density and species richness of Collembola communities in genetically modified soybean were found after GBH treatment (product not specified), due to increased root biomass of soybeans and low toxicity of the used generic GBH product containing 30% glyphosate (Chang et al. 2013). A higher abundance (Lins et al. 2007) and activity (Haughton et al. 2003; Liu et al. 2016) of Collembolans were found after the treatment with not specified GBH due to a higher nutrient availability through more available dead plant material. An increased surface activity of Collembola was also found in microcosms with seeds dressed with neonicotinoid insecticides and triazole, strobilurin and other fungicides (Zaller et al. 2016). To the best of our knowledge, only one study investigated the effects of GBH (product Montana, containing 30.8% glyphosate isopropylammonium salt) vs. the respective AI on the Collembola *F. candida* (Simões et al. 2018). There, reproduction was significantly reduced after GBH treatment but unaffected after treatment with the respective AI.

Litter decomposition is an important process integrating the biological activity of soil biota (Hättenschwiler et al. 2005). Mesofauna such as springtails are directly (by feeding) and indirectly (by regulating the activity and biomass of microorganisms) involved in litter decomposition (Cadisch and Giller 1997). Reported effects of GBH on litter decomposition are also variable. In a greenhouse experiment, no effect of GBH on litter decomposition was observed but an increase in the stabilization factor, suggesting a conversion from labile into more recalcitrant compounds (Gaupp-Berghausen et al. 2015). In contrast, other studies found no effect of GBH on litter decomposition in greenhouse (van Hoesel et al. 2017) or field studies (Casabé et al. 2007; Hagner et al. 2019).

Hence, for this study, we hypothesised that (i) GBHs have similar effects on the activity of Collembola and litter decomposition than their AIs considering that included adjuvants are chemically inert and (ii) higher SOM content in soils will increase the activity of Collembola and litter decomposition and thereby also interactively affect potential effects of GBHs or AIs. These hypotheses were tested in a factorial pot experiment in the greenhouse.

## Materials and methods

### Experimental setup

The experiment was conducted between April and July 2018 in a greenhouse of the University of Natural Resources and Life Sciences Vienna (BOKU), Austria. Experimental units were 20 l plastic pots (height 23 cm, diameter 31 cm) filled with topsoil (0–25 cm) from arable fields from two sites of the BOKU Research Farm in Groß Enzersdorf close to the city of Vienna. The study was designed as a factorial experiment using the factors treatment (GBH or respective AIs each with three levels; mechanical weeding as control), SOM content (two levels: low vs. high) and their interactions (for details, see below). The setup of the pots consisted of (3 GBH + 3 AIs + 1 control) * 2 SOM levels * 5 replicates = 70 pots that were randomly placed in the greenhouse. During the course of the experiment, average air temperature in the greenhouse was 21.3 ± 4.1 °C at natural light conditions.

Formulated GBHs included Roundup LB Plus (further called LB), Roundup PowerFlex (PF, both Monsanto Agrar Deutschland GmbH, Düsseldorf, Germany) and Touchdown Quattro (TQ, Syngenta Agro GmbH, Vienna, Austria). LB and PF were purchased in a garden shop in Vienna (Bauhaus, Vienna), and TQ was purchased in Czech Republic (VMD DROGERIE, Veselí nad Moravou).

The corresponding AIs, various salts of glyphosate (N-phosphonomethyl-glycine), i.e. glyphosate isopropylamine salt (AI of LB at 486 g l^−1^), potassium salt (AI of PF 588 g l^−1^) and diammonium salt (AI of TQ at 435 g l^−1^), were obtained from commercial sources or synthesized at the Agro-Environmental Research Institute of the National Agricultural Research and Innovation Centre, Budapest, Hungary. Glyphosate isopropylamine salt was purchased from Toronto Research Chemicals (North York, Canada), while the potassium and diammonium salts were prepared from glyphosate purchased from Sigma-Aldrich, Hungary (Budapest, Hungary). Thus, 1.66 g (9.82 mmol) of glyphosate was gradually added under continuous stirring to a cooled 0.84 ml aliquot of a 45% (w/w) aqueous potassium hydroxide solution. The mixture was stirred overnight at 4 °C, and the resultant precipitation was filtered and lyophilized to yield 1.04 g (5.02 mmol, 51.1%) of glyphosate potassium salt. Similarly, 1.66 g (9.82 mmol) glyphosate was gradually added under continuous stirring to a cooled 1.33 ml aliquot of a 28% (w/w) aqueous ammonium hydroxide solution. The mixture was stirred overnight at 4 °C, and the resultant precipitation was filtered and lyophilized to yield 1.01 g (4.97 mmol, 50.6%) of glyphosate diammonium salt.

Soil for low SOM treatment was collected from a conventionally farmed field at the Research Farm at the University of Natural Resources and Life Sciences Vienna: SOM content 3.0%, P = 73 mg kg^−1^, K = 140 mg kg^−1^, and pH (CaCl_2_) = 7.7. Soil for the factor high SOM was collected from a nearby organically farmed field: SOM content 4.1%, P = 113 mg kg^−1^, K = 234 mg kg^−1^, and pH (CaCl_2_) = 7.7. All soil properties were determined according to standard methods: SOM following ÖNORM L1080 (2013), P and K following ÖNORM L1087 (2012), and pH following ÖNORM EN15933 (2012).

Soil type was in both cases a calceric Chernozem (WRB 2014) cultivated using common crop rotations following good agricultural practice. These SOM levels reflect the average situation in conventional and organic arable farms in the region. Soil was thoroughly mixed and sieved (mesh size 1 cm), and similar amounts filled in the respective pots. The experimental soil was not sterilized and contained original soil biota. Arable soil with low SOM was treated with synthetic insecticides (active ingredients deltamethrin, pymetrozin) 3 years prior to soil sampling; no herbicides were applied on these fields for at least 5 years. Soils with high SOM were organically farmed for 25 years and not treated with synthetic insecticides or herbicides ever since.

Litter decomposition was assessed using the teabag index (TBI) following Keuskamp et al. (2013). The method works with standardized tea qualities in order to enable comparisons across ecosystems and experiments. Therefore, 4 days after setting up the pots, we buried one teabag of commercial green tea (Lipton Unilever, EAN: 8722700 05552 5) and one bag of rooibos tea (Lipton Unilever, EAN: 8722700 18,843 8) in each pot in 8 cm. Teabags of these teas consist of non-decomposable plastic material (mesh size 0.25 mm). Before insertion, teabags were dried for 1 h at 70 °C, labelled and weighed. Green tea and rooibos tea have different decomposition rates meaning that rooibos tea decomposes slower and continues when labile material in green tea has already been consumed. The stabilization process begins during the decomposition of the labile fraction of organic material. This approach has been successfully used in other ecotoxicological studies (van Hoesel et al. 2017; Zaller et al. 2018; Zaller et al. 2016).

Pots were sown with *Amaranthus retroflexus* (Amaranthaceae) as a model weed species in four rows at a row distance of 5 cm (translates to 3.9 g m^−2^, or 0.3 g pot^−1^). In arable fields of the study regions, *A. retroflexus* is a typical weed that is controlled with herbicides in conventional agriculture. Experimental plants were irrigated on 6 days per week with 0.2 l day^−1^ of tap water using a watering can, accumulating to 231 mm for the duration of the experiment (in total 80 days).

We used *F. candida* (Hexapoda: Isotomidae) as a test organism, as this species is also used for standardized ecotoxicological ISO (ISO 2014) and OECD (OECD 2009) tests. Collembolans were obtained from a certified ecotoxicology lab (ECT Oekotoxikologie GmbH, Flörsheim, Germany) and reared on activated carbon. Twenty-six days after seeding, 100 individuals of *F. candida* were added in each pot, and 7 days later, further 100 individuals were added. A transparent plastic sheet of 20-cm height was glued around the upper rim of each pot to prevent the Collembola from escaping. Once a week, chopped hay was added to the pots to provide extra food for the Collembolans. Since we worked with unsterilized soil, we assumed it contained enough fungi as food for *Folsomia* and therefore did not add extra food.

### Application of herbicides and respective active ingredients

Application of GBHs, AIs or mechanical weeding was performed 54 days after seeding, when plants were on average 22 cm high. The factor “GBHs” consisted of the three formulated herbicide products LB, PF and TQ. Factor “AIs” consisted of the respective glyphosate salts. We used dosages recommended by the responsible Austrian authority for GBHs and AIs based on active ingredients (Table 1; psmregister.baes.gv.at). GBHs/AIs were mixed with water in separate spray bottles and uniformly applied onto plants of each pot. The plants in the control pots received the same amount of water than the GBH/AI plots and were then uprooted by hand at the day of GBHs/AIs application; weeded plant material was left in the pots.

**Table 1:** Experimental treatments applied in the current experiment 54 days after seeding the model weed populations. Mechanical weeding was performed as control treatment

Treatment/product	Conc. AI	Recomm. dosage	Respective glyphosate AI
Roundup LB Plus (LB)	486 g l^−1^	5 l ha^−1^	Isopropylammonium salt (ipa)
Roundup PowerFlex (PF)	588 g l^−1^	3.75 l ha^−1^	Potassium salt (po)
Touchdown Quattro (TQ)	435 g l^−1^	5 l ha^−1^	Diammonium salt (am)
Mechanical weeding (CO)	n.a.	n.a.	n.a.

### Measurements and calculations

Collembolan activity was measured using 4 pitfall traps per pot consisting of 2-ml Eppendorf tubes (diameter 1 cm, depth 3.5 cm) filled with 1 ml of ethylene glycol and odourless detergent. Traps were carefully inserted so deep that the upper rim of the tubes was at the level of the soil surface. The traps were installed 40 days after seeding in a consistent pattern with about 10-cm distances to each other. This sampling method has been successfully applied in other experiments (Pfingstmann et al. 2019; Zaller et al. 2016). Pitfall traps were exposed for the first sampling period for 5 days and exposed for 2–3 days for the following seven sampling periods. Four samplings were conducted prior to herbicide application (days after seeding: 40–45, 45–47, 49–52, 52–53) and four after GBH/AI application (days after treatment 2–4, 7–9, 15–17, 21–23). The collected Collembola were counted and identified. The daily Collembolan activity was calculated by dividing the cumulated number of trapped Collembola by the number of days of pitfall trap exposure.

Litter decomposition was assessed using the instructions in Keuskamp et al. (2013). At the end of the experiment, after teabags were buried for 81 days, they were carefully excavated, dried at 70 °C for 48 h, cleaned and weighed again. The decomposition rate (*k*) and the stabilization factor (*S*) were calculated using the recommended hydrolysable fraction (green tea = 0.842 g g^−1^, rooibos tea = 0.552 g g^−1^). According to the protocol (www.teatime4science.org/method/stepwise-protocol/), the decomposition rate *k* (rapidly decomposed plant material with easily degradable compounds) and litter stabilization factor *S* (labile fraction, which stabilize and become recalcitrant during decomposition) were calculated. For more details on the used equations to calculate *k* and *S*, see Keuskamp et al. (2013).

Soil electric conductivity, soil moisture and soil temperature were measured twice a week using time-domain reflectometry (IMKO HD2, with calibration 01 – universal, and the moisture sensor TRIME-PICO, Ettlingen, Germany).

Plant height was measured on five randomly chosen plants of each pot to assess potential differences between treatments before GBH/AI application. At the end of the experiment (26 days after applying the treatments), *A. retroflexus* biomass in the pots was separated in dead biomass as a result of GBH/AI application or mechanical weeding and green biomass when treatments were not effective. The biomass was dried at 55 °C for 5 days and weighed afterwards.

### Statistical analyses

Statistical analyses were carried out using R version 3.3.1 (R Development Core Team 2012); *α* was set at 0.05. Normality in all data was tested via the Shapiro-Wilk test. The influence of treatment factors (GBHs, AIs and SOM levels) on Collembola activity was tested using generalized linear models (GLMs), with Poisson distribution. Data from Collembola sampling dates were tested for the complete post-application sampling period using zero inflation models, with Poisson distribution (package pscl). Not normally distributed data, such as the proportion of dead and green *A. retroflexus* biomass, *k* and *S* values from litter decomposition and Collembola activity prior to treatments were analysed using Kruskal-Wallis rank sum tests. Differences in plant height pretreatment were tested using ANOVAs. Reported data in the text are means ± SD.

## Results

No individuals of the introduced *F. candida* were caught with pitfall traps. However, three other Collembola species were caught, which were already present in the arable field soil. The species, with their average abundance across all treatment, were *Sminthurinus niger* (698 ± 415 ind. pot^−1^), *Isotoma viridis* (31 ± 12 ind. pot^−1^) and *Lepidocyrtus lanuginosus* (13 ± 7 ind. pot^−1^). As *S. niger* was by far the most abundant species and was caught in every pot, the further analyses only considered *S. niger*.

Collembolan activity across the sampling dates prior to treatment was significantly higher in high SOM pots (9 ± 8 ind. pot^−1^) than in low SOM pots (3 ± 5 ind. pot^−1^; *p* < 0.001). However, there was no significant difference in Collembola activity within the SOM levels prior to treatments (*p* = 0.439 across low SOM, *p* = 0.471 across high SOM).

Collembola activity was significantly affected by GBH, AIs and SOM levels; SOM interactively influenced effects of both GBHs and AIs (Table 2).

**Table 2:** Statistical results of effects of glyphosate-based herbicides (GBHs), their active ingredients (AIs), soil organic matter (SOM) levels and their interactions on Collembola activity, litter decomposition, abiotic soil parameters and the proportion of green plant biomass after treatment

Parameter/factor	GBHs	AIs	SOM	GBHs x SOM	AIs x SOM
Collembola act. (ind. pot^−1^)	**< 0.001**	**0.001**	**< 0.001**	**< 0.001**	**< 0.001**
Litter decomp. rate (*k*)	0.971	0.993	0.833	0.983	0.967
Litter stabilization factor (*S*)	0.751	0.844	0.857	0.833	0.954
Soil moist. (%)	**0.030**	0.784	**< 0.001**	**< 0.001**	**0.006**
Soil temp. (°C)	0.582	0.730	0.937	0.643	0.577
Soil el. cond. (dS)	0.911	0.706	0.166	0.979	0.356
Proportion green biomass (%)	**< 0.001**	**< 0.001**	0.873	0.299	0.942

*p* values from generalized linear models (GLMs). Significant effects in bold

**Fig. 1:**
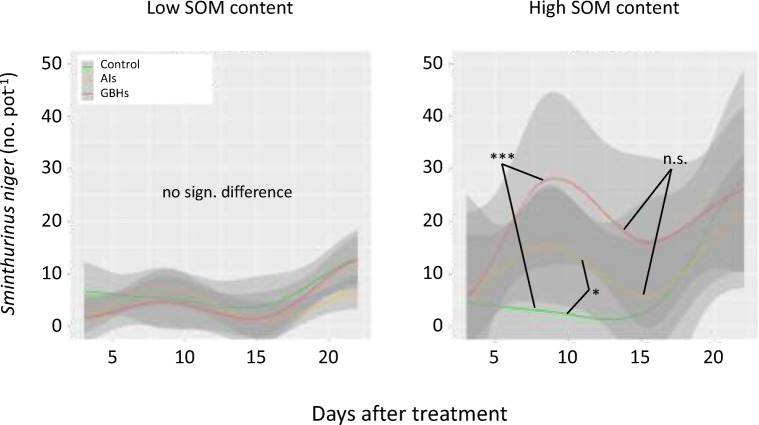
Smoothed conditional means of Collembola activity under low and high soil organic matter (SOM) after treatment with glyphosate-based herbicides (GBHs), their active ingredients (AIs) or mechanical weeding (control). Shaded areas show 95% confidence intervals. Activity is the cumulated number of Collembolans divided by days of pitfall trap exposure. Asterisks denote significant differences between treatments across the study periods: ***p < 0.001, *p < 0.05, n.s. not significant

Collembola activity was significantly lower at low SOM levels than at high SOM levels (Fig. 1). Under low SOM, Collembola activity was similar between GBHs, AIs and the control pots (Fig. 1). Under high SOM, Collembola activity was stimulated under GBH (*p* < 0.001) and AIs (*p* < 0.05; Fig. 1). There was no significant difference between GBHs and AIs under high SOM.

**Fig. 2:**
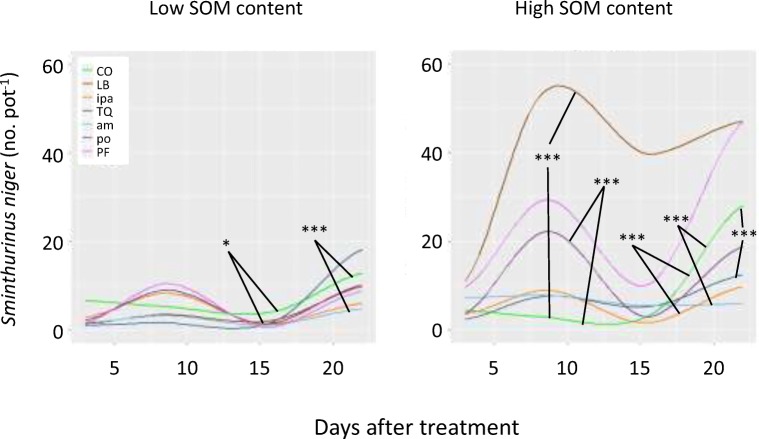
Smoothed conditional means of Collembola activity under low and high soil organic matter (SOM) after treatment the glyphosate-based herbicides Roundup LB Plus (LB), Touchdown Quattro (TQ), Roundup PowerFlex (PF) and their respective active ingredients (*ipa* isopropylammonium salt, *am* diammonium salt, *po* potassium salt); control treatment (CO) was mechanical weeding. Activity is the cumulated number of Collembolans divided by days of pitfall trap exposure. Asterisks denote significant differences between treatments across the study periods: ***p < 0.001, *p < 0.05. No asterisk means no significant difference

A detailed look at individual GBHs or AIs showed strong differences between particular GBHs but less differences between AIs (Fig. 2). Under low SOM, all substances had a negative influence on Collembola activity, which is only significantly different from control for diammonium salt (*p* < 0.001) and LB (*p* < 0.05; Fig. 2). Under high SOM soil, LB Plus and potassium salt had a significant positive influence on Collembola activity (*p* < 0.001), while isopropylammonium salt, TQ and diammonium salt had a significant negative influence (*p* < 0.01; Fig. 2). All other treatments GBH or AI were not significantly different to each other or in comparison with the control treatments (Fig. 2).

**Fig. 3:**
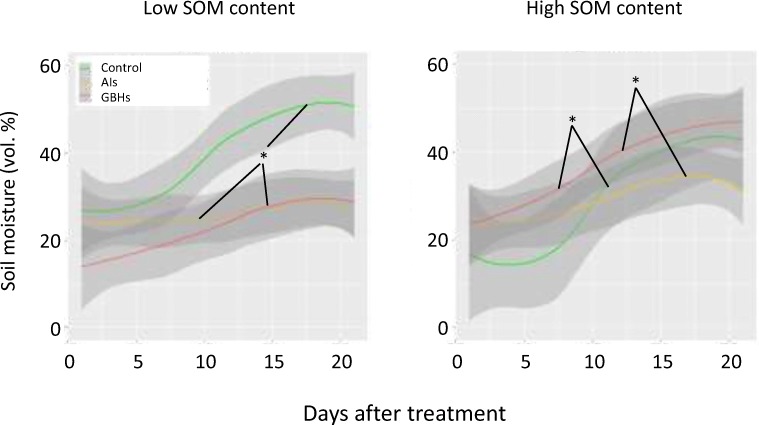
Smoothed conditional means of soil moisture under low and high soil organic matter (SOM) after treatment with glyphosate-based herbicides (GBHs), their active ingredients (AIs) or mechanical weeding (control). Shaded areas show 95% confidence intervals. Asterisks denote significant differences between treatments across the study periods: *p < 0.05. No asterisk means no significant difference

Litter decomposition rate (*k*) and stabilization index (*S*) were neither affected by GBH/AIs, nor by SOM levels (Table 2).

Soil moisture was significantly influenced by GBHs and SOM levels but unaffected by AIs (Table 2, Fig. 3). Soil moisture significantly increased under GBHs (*p* < 0.05) and high SOM content (*p* < 0.001), with significant interactive effects of SOM and GBHs (*p* < 0.001) and SOM and AIs (*p* < 0.01). Soil temperature and electrical conductivity in soil were unaffected by GBHs, AIs or SOM levels (Table 2).

Mean plant height before herbicide application was significantly higher under high SOM (across pots 23.9 ± 5.5 cm) than under low SOM (across pots 21.3 ± 5.1 cm; *p* < 0.001). However, mean plant heights were similar in pots that were later assigned to GBH or AI treatments (*p* > 0.05).

**Fig. 4:**
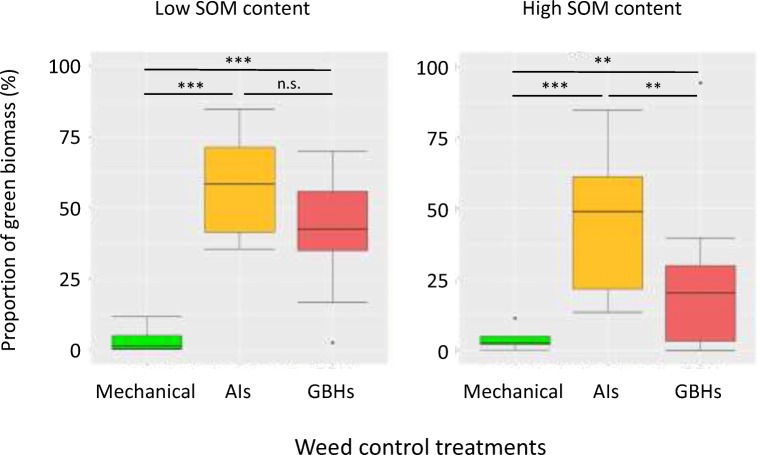
Proportion of green biomass under low and high soil organic matter (SOM) after treatment with glyphosate-based herbicides (GBHs), their active ingredients (AIs) or mechanical weeding (control). Asterisks denote significant differences between treatments across the study periods: ***p < 0.001, **p < 0.01, n.s. not significant

At harvest, proportion of green biomass was significantly influenced by GBHs, AIs or mechanical weeding, but not by SOM content (Table 2, Fig. 4). The control group had under both SOM levels the lowest amount of green biomass.

## Discussion

To the best of our knowledge, this is the first study comparing effects on Collembola and litter decomposition of three GBHs and their respective AIs under two SOM levels. The findings are interesting as ERAs of pesticides commonly mainly consider nontarget effects of AIs at one particular soil SOM level.

Initially, we wanted to assess nontarget effects using the Collembola species *F. candida*, which is a surrogate species for soil fauna in ecotoxicological studies (Ockleford et al. 2017). However, we were surprised to find no individuals of this species in our pitfall traps, although others successfully used this sampling method with this species (Buch et al. 2016). Instead, we collected many individuals of another Collembola species, *Sminthurinus niger* with our traps, and could therefore use this species for our assessment. *Sminthurinus niger* is a black, globular springtail (Hexapoda, family Katiannidae) with unclear but most likely ubiquitous distribution in many terrestrial habitats in temperate climates (Fjellberg 2007; Womersley 1931). It is also considered more surface-dwelling (Buşmachiu et al. 2015) than the mainly soil-dwelling *F. candida* (Marx et al. 2009), which would explain why there were no individuals of *F. candida* caught in the pitfall traps. Since we thoroughly homogenized the soil before distributing it among the experimental pots, we assumed an equal distribution of *S. niger* from the beginning of the experiment.

While the activity of *S. niger* was similar between GBHs, AIs and mechanical weeding in low SOM soils, it was significantly higher under GBHs/AIs in high SOM soil. The general increase in Collembola activity under high SOM was probably due to the inherently higher soil moisture as well as higher P and K contents in these soils. The higher activity of Collembolans under GBHs than under AIs or control is in contrast to our hypothesis and suggests that adjuvants contained in GBH add some additional effect to AIs.

Comparing the tested GBHs, it was seen that Roundup LB Plus and Roundup PowerFlex had the strongest stimulating effect on Collembola activity, while Touchdown Quattro had similar effects than mechanical weeding. Of the tested AIs, only potassium salt (AI of Touchdown Quattro) showed marked different effects than mechanical weeding, while the AIs isopropylammonium and diammonium salt showed similar patterns as mechanical weeding. The finding that both GBHs and AIs differed in their effects on Collembola activity suggests that other ingredients than the AIs and/or interactions with certain soil parameters might play an important role. However, these interactions are difficult to assess as the complete list of ingredients is considered a trade secret and not disclosed.

Different effects on Collembola between commercial formulation and their respective AI have also been found by others, when comparing commercial formulations of metsulfuron-methyl-based herbicides and their respective AIs (de Santo et al. 2019; de Santo et al. 2018). These studies show that the effects of the commercial formulation on the reproduction, avoidance behaviour and feeding activity of the Collembola species *Proisotoma minuta* were more detrimental than that of the respective AI alone (de Santo et al. 2019; de Santo et al. 2018).

Stimulations of Collembola activity was also observed with GBHs (Chang et al. 2013; Haughton et al. 2003; Lins et al. 2007; Liu et al. 2016). Stimulated Collembola activity after GBH application was attributed to a higher root biomass (Chang et al. 2013) or a higher amount of dead plant material and therefore a higher nutrient availability (Haughton et al. 2003; Liu et al. 2016). However, this explanation does not serve for the current experiment as mechanically weeded pots had significantly more dead plant biomass but less Collembola activity than pots treated with GBHs or AIs. Collembolans (*Sinella curviseta*) have also been shown to avoid insecticide and fungicide seed dressings (Zaller et al. 2016), and we interpret the current findings also as an avoidance behaviour of plant material and soil surface contaminated with GBHs or AIs. However, it was also shown by others that *F. candida* either avoids different GBH products (Zapp Qi 620) at recommended dosage or does not respond to other GBHs (Roundup Original, Trop, Crucial) (Niemeyer et al. 2018). The current study also highlights the importance of considering different GBHs for the same AI in risk assessment of pesticides. Generally, it is difficult to draw general behaviour patterns of Collembolans among these studies because different Collembola species, GBHs and AIs at various soil conditions were used.

Regarding the temporal dynamic of Collembola activity, we observed a peak about 9 days after GBHs or AIs application, a decrease in activity after 13 days and a further increase afterwards. These oscillations in Collembola activity are difficult to interpret and likely reflect changes in GBH/AI toxicities, population variations of Collembolans and concurrent changes in abiotic parameters. Further detailed studies would be necessary to identify the predominant driving factors. Also Lins et al. (2007) found an increased Collembola population density 10 days after an application of a not specified GBH and a decrease in densities again after 20 days. These and our findings suggest that Collembola are briefly stimulated by microbial communities that decompose certain chemical substances of herbicides, until the decomposition of the chemicals occurs, and the population comes back to normal. An initial stimulation after GBH application has also been reported for earthworms (Gaupp-Berghausen et al. 2015) or soil microorganisms (Mandl et al. 2018). Clearly, more research is needed to further elucidate the underlying mechanisms.

Among the most interesting findings of our study were the interactions between SOM and both GBHs and AIs. This aspect is commonly not addressed in ERAs where standard substrates with a given SOM level are used. The finding that GBHs and AIs did not affect Collembolan activity under low SOM but affected Collembolans under high SOM could be attributed to complex interactions between different soil properties under the two SOM levels and the amount of dead plant material on the soil surface providing shelter for Collembolans. Generally, soils with higher SOM levels usually provide more food and microhabitats and more favourable hydraulic conditions for Collembolans than low SOM soils (Filser 2002). A higher Collembola activity on soils with higher SOM is also in line with other studies (Potapov et al. 2017; Rendoš et al. 2016). However, it is also important to note that the different soil types were also taken from different fields, and high SOM soils were also higher in available P and K. Moreover, the different pesticide application history of the fields might play a role: High SOM soils were under organic cultivation for 25 years, while low SOM were conventionally managed. However, low SOM fields did not receive herbicide applications at least during the last 3 years but did receive an insecticide treatment 3 years before the current experiment was performed.

Plant litter decomposition was neither affected by GBHs, by AIs nor by SOM levels. This is in accordance with Casabé et al. (2007) and van Hoesel et al. (2017) who found no effect of GBHs on litter decomposition. Obviously, GBH or AI amounts reaching soil layers where teabags were installed (8 cm) were so small that no effect on litter decomposers was seen. In contrast, others found significant increases in the decomposition stability of plant litter due to GBH (Roundup Speed, Roundup Alphee) application (Gaupp-Berghausen et al. 2015). Certain soil microbiota might also be stimulated by GBH application (Roundup PowerFlex) leading to an increased plant litter decomposition (Mandl et al. 2018).

Soil moisture was not only affected by SOM content but also by GBH and their interactions. Also, Gaupp-Berghausen et al. (2015) found a higher soil moisture after GBH application and explained this through the lack of physiologically active, transpiring plants. However, as GBHs pots in the current experiment had more living plant mass than control pots, it could be that the green plant mass was not physiologically active any more. More studies seem necessary to also investigate abiotic soil parameters when addressing nontarget effects of GBH or AIs.

It was surprising to see that neither GBHs nor AIs readily killed our model weed population. A reason for this could be that our plants were already 22 cm high when GBHs/AIs were applied although both GBHs and AIs are considered systemic and be able to kill individuals with greater height. *Amaranthus* species have also been identified as developing resistances against glyphosate; however, so far, no resistance was reported for our study species *A. retroflexus* (Vieira et al. 2018). In our study, pots treated only with AIs had more living green biomass remaining than GBHs again indicating that adjuvants mixed to herbicide formulations are not chemically inert. Mechanical weeding showed most effective result in controlling model weeds.

## Conclusions

We conclude that commercial GBHs differ in their effects on Collembola and capability to kill plants compared to their respective AIs and that these effects are influenced by soil SOM levels. This is a new finding important to be taken into account in pesticide ERAs which currently mainly consider nontarget effects of AIs and not of formulated products. Side effects of formulants obviously cannot be covered in ERA of AIs in the dual level pesticide registration system in the European Union (EU) with AIs authorized at EU level and formulated products approved at Member State (MS) level. Detrimental effects of formulants can be currently identified at MS level, yet more rigorous registration criteria should be exerted to identify possible toxicity of formulation additives. Another possible solution to this problem could be if formulants, adjuvants and additive substances were required to be individually registered, similarly to AIs, at EU level. Yet another solution could be if pesticide registration were rendered more similar to that of veterinary substances (Klátyik et al. 2017; Székács and Darvas 2018). In contrast to the prevailing view that herbicides specifically affect only plants and certain microorganisms, our findings corroborate other studies documenting various nontarget effects on soil fauna. To what extent an increased or decreased surface activity of Collembola is affecting their population development remains to be investigated. In any case, more surface active Collembolans are more prone to predators such as spiders (Pfingstmann et al. 2019). Our results also suggest that other Collembola species than the commonly used *F. candida* might be suitable surrogate species for ERAs. Clearly, more investigations considering long-term effects, reproduction and cascading effects at different trophic levels seem necessary (Brühl and Zaller 2019). Based on our findings of interactive effects between SOM content and GBHs/AIs, we suggest that ERAs also consider potential nontarget effects under different soil characteristics.
